# Adenosine A_2A_ Receptors Activation Facilitates Neuromuscular Transmission in the Pre-Symptomatic Phase of the SOD1(G93A) ALS Mice, but Not in the Symptomatic Phase

**DOI:** 10.1371/journal.pone.0104081

**Published:** 2014-08-05

**Authors:** Filipe Nascimento, Paula A. Pousinha, Alexandra M. Correia, Rui Gomes, Ana M. Sebastião, Joaquim A. Ribeiro

**Affiliations:** 1 Institute of Pharmacology and Neurosciences, Faculty of Medicine, University of Lisbon, Lisbon, Portugal; 2 Unit of Neurosciences, Instituto de Medicina Molecular, University of Lisbon, Lisbon, Portugal; 3 National Museum of Natural History and Science, University of Lisbon, Lisbon, Portugal; 4 Faculty of Sciences, University of Lisbon, Lisbon, Portugal; University of Sydney, Australia

## Abstract

Amyotrophic Lateral Sclerosis (ALS) is a neurodegenerative disease leading to motor neuron dysfunction resulting in impairment of neuromuscular transmission. A_2A_ adenosine receptors have already been considered as a potential therapeutical target for ALS but their neuromodulatory role at the neuromuscular junction in ALS remains to be clarified. In the present work, we evaluated the effects of A_2A_ receptors on neuromuscular transmission of an animal model of ALS: SOD1(G93A) mice either in the pre-symptomatic (4–6 weeks old) or in the symptomatic (12–14 weeks old) stage. Electrophysiological experiments were performed obtaining intracellular recordings in Mg^2+^ paralyzed phrenic nerve-hemidiaphragm preparations. Endplate potentials (EPPs), quantal content (q. c.) of EPPs, miniature endplate potentials (MEPPs) and giant miniature endplate potential (GMEPPs) were recorded. In the pre-symptomatic phase of the disease (4–6 weeks old mice), the selective A_2A_ receptor agonist, CGS 21680, significantly enhanced (p<0.05 Unpaired *t*-test) the mean amplitude and q.c. of EPPs, and the frequency of MEPPs and GMEPPs at SOD1(G93A) neuromuscular junctions, the effect being of higher magnitude (p<0.05, Unpaired *t*-test) than age-matched control littermates. On the contrary, in symptomatic mice (12–14 weeks old), CGS 21680 was devoid of effect on both the amplitude and q.c. of EPPs and the frequency of MEPPs and GMEPPs (p<0.05 Paired *t*-test). The results herein reported clearly document that at the neuromuscular junction of SOD1(G93A) mice there is an exacerbation of A_2A_ receptor-mediated excitatory effects at the pre-symptomatic phase, whereas in the symptomatic phase A_2A_ receptor activation is absent. The results thus suggest that A_2A_ receptors function changes with ALS progression.

## Introduction

Amyotrophic Lateral Sclerosis (ALS) is an adult-onset progressive neurodegenerative disease characterized by the selective loss of motor neuron function leading to muscle atrophy and weakness. After symptomatic onset disease progression lasts 4 to 5 years and patients ultimately die due to bulbar failure. Most of the diagnosed cases carry an unknown genetic link (sporadic ALS) and a few (5–10%) are related to known mutations in specific proteins (familial ALS). Both present similar pathological and clinical features [1], [2]. The first gene associated with the inherited form of the disease was the SOD1 gene encoding for the superoxide dismutase 1 enzyme which accounts for 20% of the familial forms of ALS [3]. This led to the design of the first animal model of ALS, the SOD1(G93A) mouse, which currently is the most used and well characterized rodent model for this disease [4]. Neuromuscular dysfunction at symptomatic SOD1(G93A) mice has been reported [5], [6]. We recently showed that the SOD1(G93A) mice neuromuscular transmission impairment starts long before symptomatic onset [7].

Adenosine is a key neuromodulator with implications in pathological conditions [8]. At the neuromuscular junction it can act on both A_1_ and A_2A_ adenosine receptors, fine-tuning acetylcholine (ACh) release [9]. A_2A_ receptors are known to have a neuroprotective role in some pathological conditions [8] and have been considered as a potential therapeutical target for ALS [10]–[12]. Some contradictory reports in the literature can however be found [10], [11] highlighting the need for an evaluation of the influence of A_2A_ receptors in ALS models where disease progression and neuromuscular transmission impairment can be taken into account.

Given the unexplored role of A_2A_ receptors at the neuromuscular junction in ALS, and considering that the neuromuscular transmission in the SOD1(G93A) mice starts to present alterations long before symptoms onset [7], we considered of interest to evaluate A_2A_ receptor effects on neuromuscular transmission, in both pre-symptomatic (4–6 weeks old) and symptomatic (12–14 weeks old) SOD1(G93A) ALS mice. The results now reported show that the role of A_2A_ receptors at the motor nerve terminals, changes upon ALS progression. In the pre-symptomatic phase the A_2A_ receptor-mediated excitatory effects on neuromuscular transmission are exacerbated, probably acting as a compensatory mechanism towards delaying disease progression, whereas in the symptomatic phase the A_2A_ receptor excitatory action disappears.

## Methods

### Ethics statement

This study was performed in accordance with the European Community guidelines (Directives 86/609/EU and 2010/63/EU, Recommendation 2007/526/CE, European Convention for the Protection of Vertebrate Animals used for Experimental or Other Scientific Purposes ETS 123/Appendix A) and Portuguese Laws on Animal Care (Decreto-Lei 129/92, Portaria 1005/92, Portaria466/95, Decreto-Lei 197/96, Portaria 1131/97). All the protocols carried in this study were under approval of the Portuguese National Authority (General Direction of Veterinary) and the Ethics Committee of the Instituto de Medicina Molecular of the Faculty of Medicine, University of Lisbon, Lisbon, Portugal.

### Animals

Transgenic B6SJL-TgN (SOD1-G93A)1Gur/J males (Jackson Laboratory, No. 002726) overexpressing the human SOD1 gene carrying a glycine to alanine point mutation at residue 93 (G93A) [4] and wild-type B6SJLF1/J females were purchased from The Jackson Laboratory (Bar Harbor, ME, USA) and were breed at IMM rodent facilities where a colony was established. Mice were maintained on a background B6SJL by breeding SOD1(G93A) transgenic males with non-transgenic females in a rotational scheme. Males were crossed with non-transgenic females because transgenic females are infertile. F1 offspring was used in all experiments. Progeny was no longer used in breeding to avoid mSOD1 gene copy number loss and therefore deviation from ALS phenotype [4]. SOD1(G93A) mice were used to study pre-symptomatic (4–6 weeks old) and symptomatic (12–14 weeks old) phases of the disease. 4–6 and 12–14 weeks old wild type (WT) animals served as controls. Both male and female mice were used. The proportion of male and female mice was about the same in the WT (18 males and 19 females in total) and SOD1(G93A) mice (20 males and 17 females in total) groups. Furthermore, using the same mice strain (B6SJL-Tg(SOD1-G93A)1Gur/J) no gender influences over the intrinsic features of neuromuscular transmission have been detected [7], though gender differences could be detected in other mice strains (B6.Cg-Tg-(SOD1-G93A)1Gur/J) [6].

Littermates were identified by dermal ear punching and divided into cages by gender. The ear tissue was used to genotype the animals by polymerase chain reaction (PCR) [3]. Animals were housed 4–5 mice/cage, under a 12 h light/12 h dark cycle, and received food and water *ad libitum*.

### Electrophysiological recordings

Animals were anaesthetised using halothane and rapidly decapitated. Both right and left phrenic-nerve attached to the hemidiaphragm muscle were isolated. One preparation was placed and stretched in a 3 mL Perspex chamber continuously perfused via a roller pump (3 mL.min^−1^) with a physiologic saline solution (Krebs and Henseleit solution, see Drugs section) under continuous oxygenation. The other phrenic-nerve hemidiaphragm preparation was kept in a beaker with an oxygenated saline solution before being set up the recording chamber. Since no functional differences were found between right and left phrenic nerve-hemidiaphragm muscles, different protocols were carried in each preparation.

Intracellular recordings were performed in the conventional way [13]–[15]. The phrenic-nerve was stimulated supramaximally by a suction electrode (Cu/Cu^2+^) connected to a S48 square pulse stimulator (Grass Tecnologies, West Warwick, RI, USA). Stimuli were applied in a low frequency of 0.5 Hz with a current duration of 20 µs. The reference electrode was an Ag-AgCl pellet placed in the bath. The recording electrode was a glass microelectrode filled with KCl (3 M) with resistance between 15–40 MΩ inserted into the motor endplate. A Digidata 1440A digitizer (Molecular Devices, Sunnyvale, CA, USA), designed to work with the Axoclamp 2B amplifier (Molecular Devices, Sunnyvale, CA, USA), performed data acquisition, allowing continuous monitoring and digital storage of evaluated parameters with adequate software (pCLAMP 10.3, Molecular Devices, Sunnyvale, CA, USA).

Endplates with a resting potential between –65 to –85 mV were chosen for experiment. Resting voltage was stable throughout all experiments with less than 5% variation of its initial value. Endplate Potentials (EPPs) amplitude was assessed as the average amplitude of 60 consecutive EPPs (with amplitudes ranging between 1 mV to 5 mV). To evaluate the percentage of the drug effect, the mean averaged EPP amplitudes in the last 10 minutes before adding any drug (control) was compared with the mean averaged EPP amplitudes from the last 10 minutes of drug perfusion (treatment). The quantal content (q. c.) of EPPs was calculated as the ratio between the mean EPP amplitude and the mean Miniature Endplate Potential (MEPP) amplitude acquired during the same period with the same resting membrane potential. MEPPs were recorded in gap-free intervals of 100 seconds before adding the drug and at the end of drug perfusion. MEPP detection threshold was set between 0.2 mV and 1 mV [7]. MEPP amplitude was defined as the mean of all spontaneous events and the frequency as the number of events registered during the 100 seconds. The minimum Giant Miniature Endplate Potential (GMEPP) threshold amplitude was set in 1 mV [7]. This indirect measure of spontaneous activity synchronism was analyzed as the frequency of giant events in the 100 seconds gap-free acquisition mode and the mean amplitude as the average of GMEPPs magnitude in the same interval. Only if GMEPP frequency was higher than 0.04 s^−1^ before adding the drug, the percentage of effect was considered for analysis. Evoked activity was analyzed with Clampfit software (Molecular Devices, Sunnyvale, CA, USA) and spontaneous events with Mini-Analysis software (Synaptosoft Inc., Decatur, GA, USA). Whenever perfusing two drugs, the % change was calculated by comparing acquired values with the ones obtained from the first drug perfused (considered then as control).

### Drugs

The bathing solution was modified from Krebs and Henseleit [16] (NaCl 117 mM; KCl 5 mM; NaHCO_3_ 25 mM; NaH_2_PO_4_ 1.2 mM; glucose 11 mM; CaCl_2_ 2.5 mM; MgCl_2_ 1.2 mM; pH 7.4) continuously gassed with 95% O_2_ and 5% CO_2_ kept at room temperature (22–25°C). Muscle twitch was prevented by increasing [Mg^2+^] to 18.5–19.5 mM in 4–6 weeks old animals and 20.0–22.0 mM in 12–14 weeks old mice. This strategy reduces the q. c. of EPPs but preserves the main features of neuromuscular transmission [13].

Drugs used were: 2-*p*-(2-carboxyethyl) phenethylamino]-5′-N-ethylcarboxamido adenosinehydrochloride (CGS 21680) and 5-Amino-7-(2-phenylethyl)-2-(2-furyl)-pyrazolo(4,3-*e*)-1,2,4-triazolo(1,5-*c*) pyrimidine (SCH 58261). Stock solutions (5 mM) were made in dimethyl sulfoxide. To avoid compound precipitation aliquots were kept frozen at –20°C until used. Dimethyl sulfoxide was devoid of effect in the performed experiments like previously reported [13].

### Statistical analysis

Data are presented as mean ± standard error of the mean in each group, which n corresponds to the number of animals used (1 fiber per mouse).

Student’s *t*-test for independent samples (Unpaired *t*-test) was used to compare drug effect between two groups. One way analysis of variance (ANOVA) was applied whenever comparing more than 2 means. If p<0.05, Tukey’s pos-test was applied to compare drug-induced changes between different groups. Student’s *t*-test for paired samples (Paired *t*-test) was used to compare obtained measurement with the control parameter before adding the drug (e.g. mean EPP amplitude before drug perfusion). Values of p<0.05 were considered to represent statistically significant differences.

## Results

### In pre-symptomatic SOD1(G93A) mice the excitatory A_2A_ receptor-mediated effects on neuromuscular transmission are exacerbated

**Figure 1:**
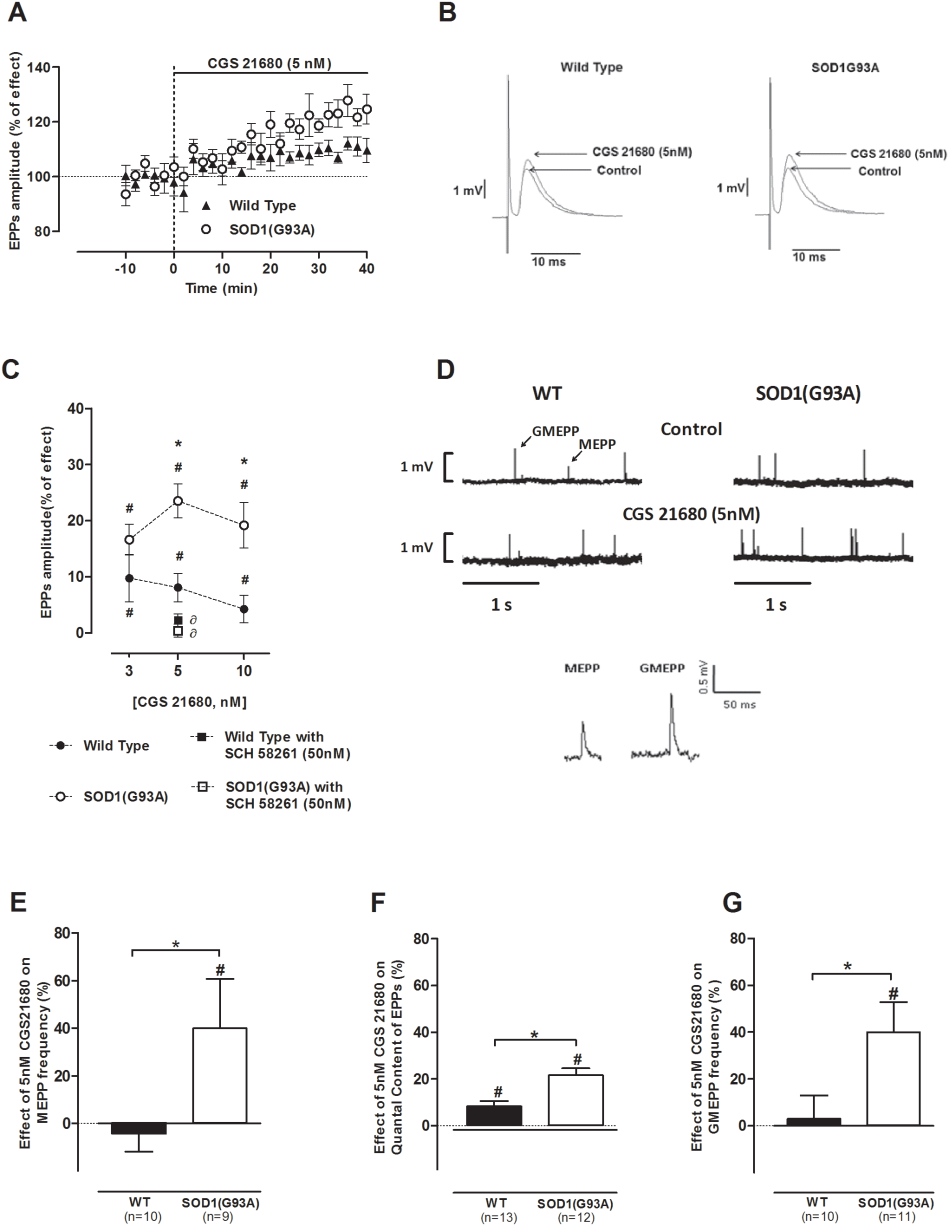
CGS 21680 facilitation of evoked activity is exacerbated in pre-symptomatic mice; (A) representative time-course change of mean EPP amplitude throughout CGS 21680 (5 nM) perfusion and (B) representation of EPP amplitude increase in 4–6 weeks old WT (n = 5) and pre-symptomatic mice (n = 10) upon A_2A_ receptor activation (CGS 21680 at 5 nM); (C) concentration-response changes in mean EPP amplitude in the presence of CGS 21680 (3 nM: n = 7, WT, n = 7, SOD1G93A; 5 nM: n = 14, WT, n = 13, SOD1G93A; 10 nM: n = 7, WT, n = 5, SOD1G93A) whose effect was blocked by SCH 58261 at 50 nM (n = 5, WT, n = 4, SOD1G93A); (D) raw recording of spontaneous release fluctuations from a 4–6 weeks old WT and pre-symptomatic SOD1G93A neuromuscular junction promoted by CGS 21680 (5 nM); effect of CGS 21680 (5 nM) perfusion regarding (E) MEPP frequency (n = 10, WT, n = 9, SOD1(G93A), (F) quantal Content of EPPs (n = 13, WT, n = 12, SOD1(G93A)) and (G) GMEPP frequency (n = 10, WT, n = 11, SOD1(G93A)) in pre-symptomatic SOD1(G93A) mice and respective healthy controls; *p<0.05 Unpaired *t*-test; ^∂^p<0.05 one-way ANOVA with Tukey’s pos-hoc; ^#^p<0.05 Paired *t*-test (as compared with control value before drug perfusion); control corresponds to 100% in all cases.

Though the selective A_2A_ receptor agonist CGS 21680 [17] has been extensively used in research, namely at the neuromuscular junction [9], [13], [18], there are only few studies reporting its effects at the mouse neuromuscular junction, the existing being on K^+^-evoked ACh-release (e.g. [19]). We performed a dose-response study using 3, 5 to 10 nM of CGS 21680 in both pre-symptomatic SOD1(G93A) mice and their age-matched healthy controls. Figure 1A illustrates the time-course changes of mean EPP amplitude in the presence of CGS 21680 (5 nM). It clearly shows an exacerbation of the facilitatory effect of the A_2A_ receptor agonist on EPP amplitude, in the pre-symptomatic SOD1(G93A) mice, a finding also illustrated in figure 1B. The difference between the two groups started to be evident 20 min after drug perfusion. As illustrated in Figure 1C, all the tested concentrations enhanced the mean amplitude of EPPs, when compared to the value measured before drug perfusion (p<0.05, Paired *t*-test). To evaluate the role of A_2A_ receptors on neuromuscular transmission, while comparing the effect of CGS 21680 on the mean amplitude of EPPs recorded in both groups of animals, it could be concluded that at 3 nM there were no significant differences between groups (p>0.05, Unpaired *t*-test) while at 5 nM and 10 nM the facilitation caused by CGS 21680 on EPPs amplitude was significantly higher in pre-symptomatic SOD1(G93A) mice (5 nM: n = 13; 10 nM: n = 5; p<0.05, Unpaired *t*-test), when compared to the WT group (5 nM: n = 14; 10 nM: n = 7). Since the difference between groups was already pronounced at 5 nM, we decided to use this concentration in the remaining experiments. To exclude potential unspecific effects of CGS 21680, we evaluated the effect of this drug in the presence of the selective A_2A_ receptor antagonist, SCH 58261 (50 nM) [20]. The blockade of A_2A_ receptors did not change the mean amplitude of EPP in both groups of animals (data not shown, n = 5 for controls and n = 6 for SOD1G93A; p>0.05, Paired *t*-test) and effectively prevented the facilitatory effects of CGS 21680 (5 nM) (Figure 1C, n = 4 for controls and n = 6 for SOD1G93A; p<0.05, one-way ANOVA followed by Tukey’s pos-hoc). These results suggest that in the present experimental conditions A_2A_ receptors are not tonically activated by endogenous adenosine and, also, that the effect of CGS 21680 (5 nM) results from specific A_2A_ receptor action upon neuromuscular transmission.

To evaluate changes in the q. c. of EPPs, MEPPs and EPPs were recorded simultaneously. As illustrated in Figure 1D–E, when tested in WT animals, CGS 21680 (5 nM) was devoid of effect on both MEPPs amplitude and frequency (n = 10; p>0.05, Paired *t*-test). However, when applied to pre-symptomatic SOD1(G93A) neuromuscular junctions it caused a significant increase in the frequency of MEPPs, without changing its average amplitude (n = 9; p<0.05, Unpaired *t*-test). As it occurred for evoked changes in EPPs amplitude, the A_2A_ receptor-mediated facilitatory effect on the mean frequency of MEPPs was more pronounced in pre-symptomatic SOD1(G93A) mice than in its age-matched healthy controls (Figure 1E). Also, SCH 58261 (50 nM), *per se*, did not significantly change MEPPs frequency in both studied animal groups (data not shown, n = 8 for controls and n = 4 for SOD1(G93A); p>0.05 Paired *t*-test), while preventing the facilitatory action of the A_2A_ receptor agonist upon MEPP frequency in the pre-symptomatic SOD1(G93A) mice (n = 4, p<0.05, one-way ANOVA followed by Tukey’s pos-hoc). Regarding the q. c. of EPPs (Figure 1F) we observed that CGS 21680 (5 nM) caused a significantly higher facilitation in pre-symptomatic SOD1(G93A) mice (n = 12) than in its age-matched healthy mice (n = 13; p<0.05, Unpaired *t*-test); this effect was prevented by SCH 58261 (50 nM; n = 4 for controls and n = 6 for SOD1(G93A); p<0.05, one-way ANOVA followed by Tukey’s pos-hoc).

GMEPPs arise from intracellular Ca^2+^ disturbances resulting in a non-evoked “constitutive” secretion leading to abnormal spontaneous events at mammalian neuromuscular junctions [21], [22] and pre-symptomatic SOD1(G93A) mice present higher frequency of GMEPPs when compared to controls [7]. Considering the role of adenosine receptors in Ca^2+^ modulation [23], [24], the effect of A_2A_ receptor activation, with its selective agonist, on the amplitude and frequency of giant spontaneous events was also evaluated. As illustrated in Figure 1D and 1G, CGS 21680 (5 nM) caused a significant increase in mean frequency of GMEPPs in pre-symptomatic SOD1(G93A) mice (n = 11; p<0.05, Unpaired *t*-test), whereas in the WT group it was devoid of effect (n = 10, p>0.05, Paired *t*-test). SCH 58261 (50 nM), *per se*, did not change the mean frequency of GMEPPs (data not shown, n = 7 for controls and n = 5 for SOD1(G93A)) and effectively prevented the facilitatory effect caused by 5 nM CGS 21680 in pre-symptomatic SOD1(G93A) mice (n = 5; p<0.05, one-way ANOVA). GMEPPs amplitude remained unchanged upon CGS 21680 (5 nM) perfusion and no statistical difference was found between the studied groups of animals.

Together, the results suggest that the A_2A_ receptor-mediated facilitatory effects on neuromuscular transmission are exacerbated in the pre-symptomatic phase of the disease.

### In symptomatic SOD1(G93A) mice the excitatory A_2A_ receptor-mediated effects on neuromuscular transmission are absent

**Figure 2:**
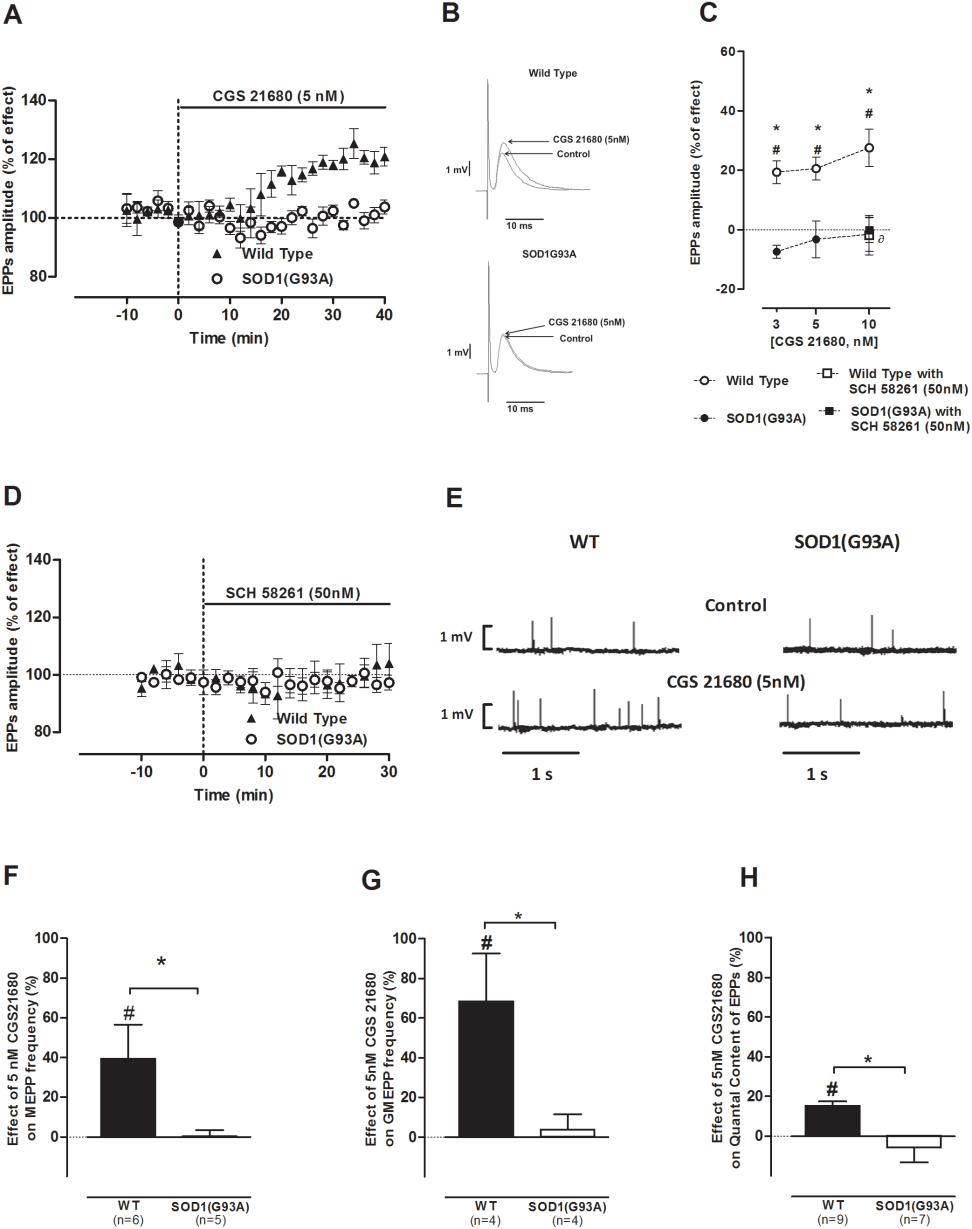
A_2A_ receptor modulation is lost in symptomatic SOD1(G93A) mice endplates; (A) representative average time-course of mean EPP amplitude change during CGS 21680 (5 nM) bathing and (B) illustrative mean EPP profile facilitation in 12–14 weeks old control (n = 6) and symptomatic mice (n = 6); (C) dose-response alterations in mean EPP amplitude by CGS 21680 (3 nM: n = 8, WT, n = 10, SOD1G93A; 5 nM: n = 10, WT, n = 7, SOD1G93A; 10 nM: n = 11, WT, n = 7, SOD1G93A) were blocked by SCH 58261 at 50 nM in WT mice (n = 4, WT, n = 4, SOD1G93A); (D) SCH 58261 (50 nM) did not affect evoked activity throughout data acquisition (n = 4, WT, n = 7, SOD1G93A); (E) raw recording of spontaneous release variations from a 12–14 weeks old WT and symptomatic SOD1G93A endplate upon CGS 21680 (5 nM) perfusion; effect of A_2A_ receptor activation by CGS 21680 (5 nM) on (F) MEPP frequency (n = 6, WT, n = 5, SOD1(G93A)) (G) GMEPP frequency (n = 4, WT, n = 4, SOD1(G93A)) and (H) quantal content of EPPs (n = 9, WT, n = 7, SOD1(G93A)); *p<0.05 Unpaired *t*-test; ^∂^p<0.05 one-way ANOVA with Tukey’s pos-hoc; ^#^p<0.05 Paired *t*-test (as compared with control value before drug perfusion); control corresponds to 100% in all cases.

As in the pre-symptomatic phase of the disease, we performed a concentration-response study using the same concentrations (3, 5 and 10 nM) of CGS 21680 in both symptomatic SOD1(G93A) mice and their age-matched (12–14 weeks old) healthy controls. Figure 2A shows the time course of mean EPP amplitude changes in the symptomatic SOD1(G93A) mice throughout CGS 21680 (5 nM) perfusion. Figure 2B represents the profile of mean EPP amplitude changes in WT and symptomatic mice by 5 nM of CGS 21680. As expected, all the tested concentrations enhanced the mean averaged amplitude of EPPs in WT mice, when compared to the measured value before drug perfusion (Figure 2C; 3 nM: n = 8; 5 nM: n = 10; 10 nM: n = 11; p<0.05, Paired *t*-test). Remarkably, when applied to the symptomatic SOD1(G93A) neuromuscular junctions, none of the tested concentrations modified the amplitude of EPPs (Figure 2C; 3 nM: n = 10; 5 nM: n = 7; 10 nM: n = 7; p>0.05, Paired *t*-test). As illustrated in Figure 2D, SCH 58261 (50 nM) was devoid of effect in the mean EPP amplitude in both groups (WT: n = 4; SOD1(G93A): n = 7; p>0.05 Paired *t*-test), suggesting the absence of tonic activation of the A_2A_ receptor agonist in symptomatic SOD1(G93A) mice as well in WT mice.

As illustrated in figures 2E, 2F and 2G, CGS 21680 (5 nM) caused an increase on MEPPs (n = 6) and GMEPPs frequency in WT mice (n = 4; p<0.05, Paired *t*-test) but when applied to symptomatic SOD1(G93A) neuromuscular junctions it was devoid of effect on MEPPs (n = 5) and GMEPP frequency (n = 4; p<0.05, Paired *t*-test). MEPPs amplitude remained unchanged in the presence of the A_2A_ receptor agonist in both groups (p>0.05, Unpaired t-test). In relation to the q. c. of EPPs (Figure 2H), there was a significant increase in the q. c. of EPPs in 12–14 weeks old healthy mice, upon CGS 21680 (5 nM) perfusion (n = 9; p<0.05, Unpaired t-test), which was prevented by SCH 58261 (50 nM; n = 4; p<0.05, one-way ANOVA followed by Tukey’s pos-hoc). In contrast, the q. c. of EPPs was not modified by CGS 21680 (5 nM) perfusion in symptomatic SOD1(G93A) mice (n = 7; p>0.05 Paired *t*-test).

### Comparison between the effect of A_2A_ receptors activation at SOD1(G93A) neuromuscular junctions upon disease progression

**Figure 3:**
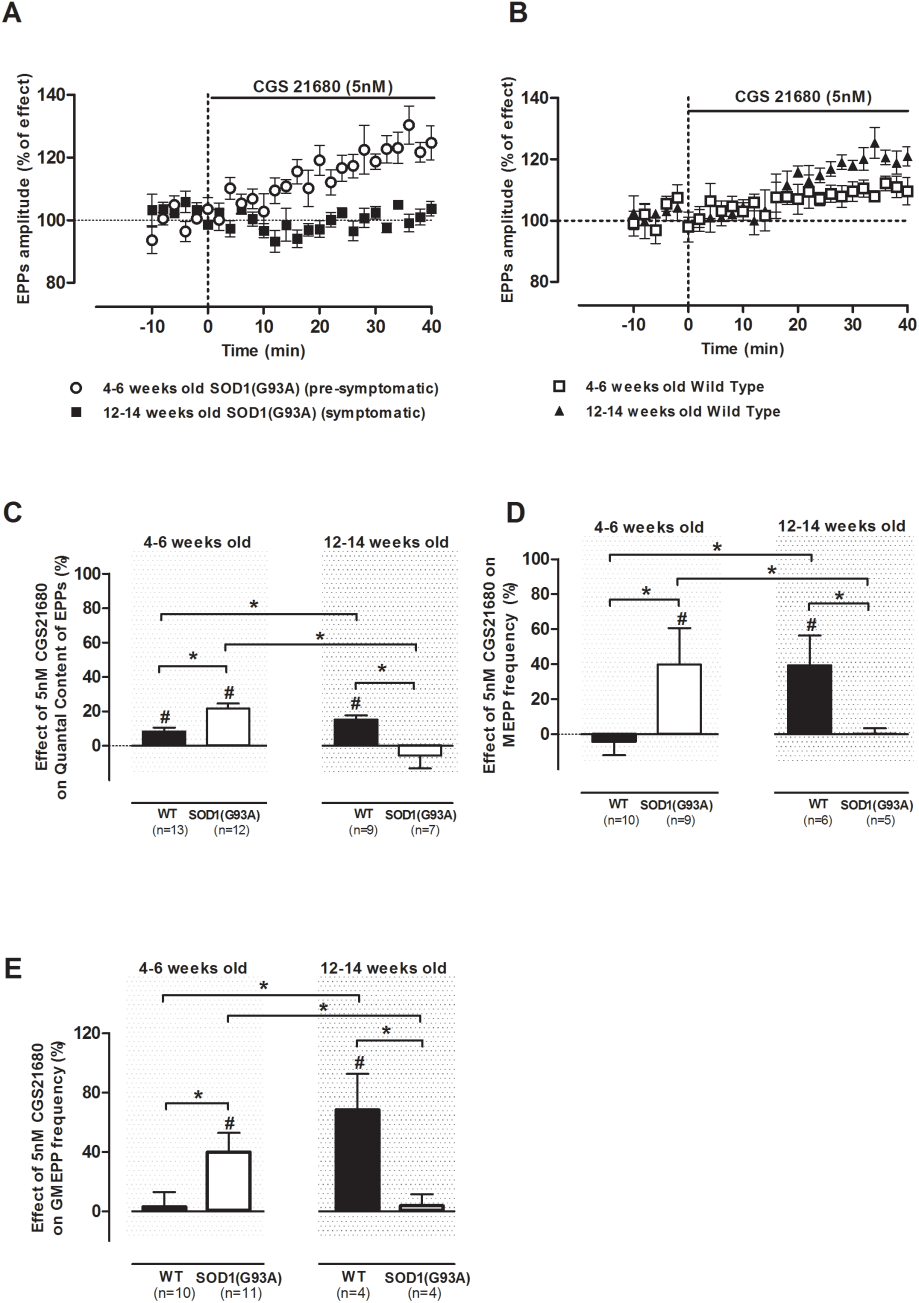
Comparison of A_2A_ receptor function upon disease progression in SOD1(G93A) mice and healthy controls; average time course of mean EPP amplitude facilitation by CGS 21680 (5 nM) in (A) pre-(n = 10) and symptomatic (n = 6) SOD1(G93A) rodents and (B) 4–6 weeks (n = 5) and 12–14 weeks (n = 6) old WT mice; effect of CGS 21680 perfusion at 5 nM on: (C) quantal content of EPPs (4–6 weeks old: n = 13, WT, n = 12, SOD1(G93A); 12–14 weeks old: n = 9, WT, n = 7, SOD1(G93A)); (D) MEPP frequency (4–6 weeks old: n = 10, WT, n = 9, SOD1(G93A); 12–14 weeks old: n = 6, WT, n = 5, SOD1(G93A)); and (E) GMEPP frequency (4–6 weeks old: n = 10, WT, n = 11, SOD1(G93A); 12–14 weeks old: n = 4, WT, n = 4, SOD1(G93A)) in both phases of the study from SOD1(G93A) mice and respective healthy controls; *p<0.05 Unpaired *t*-test; ^∂^p<0.05 one-way ANOVA with Tukey’s pos-hoc; ^#^p<0.05 Paired *t*-test (as compared with control value before drug perfusion); control corresponds to 100% in all cases.

To allow the assessment of the role of A_2A_ receptors throughout ALS progression, in Figure 3 are compared the effects of CGS 21680 (5 nM) in the pre-symptomatic and symptomatic SOD1(G93A) mice. Age-matched healthy controls were also subject of comparison to evaluate the maturation-associated alterations of neuromuscular transmission in physiological conditions. Figure 3A shows the superimposed time-course profiles of mean EPP amplitude change throughout CGS 21680 (5 nM) perfusion in pre-symptomatic and symptomatic SOD1(G93A) neuromuscular junctions. By this figure one can find that the role of A_2A_ receptors dramatically changes with disease progression. It is concluded that the A_2A_ receptor selective agonist induced a significantly higher enhancement of EPPs amplitude in 12–14 weeks old WT than in 4–6 weeks old control animals (p<0.05 Unpaired *t*-test) (Figure 3B), which was accompanied by a significant increase in the q. c. of EPPs (p<0.05, Unpaired *t*-test) (Figure 3C). Interestingly, the effect of CGS 21680 (5 nM) in pre-symptomatic SOD1(G93A) animals (4–6 weeks old) is similar to its effect in the 12–14 weeks old WT controls. This might be related to an ALS-associated early maturation process at the neuromuscular junction, as previously suggested [7].

In relation to the effect of A_2A_ receptor activation on MEPPs and GMEPPs frequency (figures 3D and 3E), there are some similarities between the pre-symptomatic SOD1(G93A) mice and the 12–14 weeks old wild-type controls. For example, the changes on the frequency of MEPPs and GMEPPs caused by A_2A_ receptor activation, observed in pre-symptomatic SOD1(G93A) mice were not statistically different from the values recorded in the 12–14 weeks old wild type controls (p<0.05 Unpaired *t*-test).

## Discussion

The main finding of the present work was that the role of adenosine A_2A_ receptors at the neuromuscular junction of the ALS SOD1(G93A) mouse model changes with disease progression. In the pre-symptomatic phase, the magnitude of the excitatory effects on neuromuscular transmission, caused by A_2A_ receptor, is enhanced compared to age-matched controls. In contrast, in the symptomatic SOD1(G93A) mice, the A_2A_ receptor-mediated facilitation is absent.

The enhancement of neuromuscular transmission caused by the selective A_2A_ receptor agonist, CGS 21680, results from an increase in the evoked release of ACh, since it increased the q. c. of EPPs without affecting the average amplitude of MEPPs recorded concomitantly. It is known that the activation of the adenosine A_2A_ receptors induces an enhancement of neuromuscular transmission, which is hardly reversible [9], [13] and apparently more robust in 3–4 weeks old rats [13] than in 4–6 weeks old mice (present work). Interestingly, the A_2A_ receptor signaling is apparently lost at the neuromuscular junction of aged (70–80 weeks old) rats as it is in the symptomatic SOD1(G93A) mice (present work), suggestive of an a disease induced early-ageing of A_2A_ receptor influence upon neuromuscular transmission. The reason for the hardly reversible adenosine A_2A_ receptor-mediated action might be the transducing system operated by the receptor, which binds to G-protein coupled receptors [25] involving cyclic AMP formation and Protein kinase A (PKA) activation [26] with subsequent protein phosphorylation, causing a long-lasting increase in synaptic strength. In addition, multiple interactions of A_2A_ receptors with other proteins have been described both in peripheral and central nervous system [8]. At the neuromuscular junction, A_2A_ receptors are known to interact with adenosine A_1_ receptors [13], [18], [27], presynaptic nicotinic autofacilitatory receptors [28], tyrosin receptor kinase B (TrkB) [29] or calcitonin gene-related peptide [30]. Furthermore, SOD1(G93A) mice pathogenesis is characterized by increased oxidative stress [2] and A_2A_ receptors present redox-sensitive synchronizing action at the neuromuscular synapse [31]. Interestingly, in a recent work from our team where neuromuscular transmission of the SOD1(G93A) mouse was studied [7], it was show that ACh release at the neuromuscular junction is enhanced in the pre-symptomatic phase of the disease, since the average amplitude of EPPs recorded in the SOD(G93A) mice during the pre-symptomatic phase (4–6 weeks old) was similar to the values obtained in the healthy control group (12–14 weeks-old). Interestingly, the levels of brain-derived neurotrophic factor (BDNF) are strongly increased in post-mortem muscle samples of early phase of ALS patients [32]. It is known that A_2A_ receptors, at motor nerve terminals, trigger the action of BDNF [29], which enhances transmitter release at developing neuromuscular junctions [33], improving neuromuscular transmission in the adult rat diaphragm [34] and facilitating synaptic efficacy by increasing presynaptic depolarization at the neuromuscular junction [35]. BDNF is also important for maintenance of ACh receptor clustering in the endplate [36], [37]. Whether the enhancement by A_2A_ receptor in the pre-symptomatic phase of the disease, could account for the potentiation of endogenous BDNF actions that might occur at the neuromuscular junction, therefore, enhancing synaptic transmission and compensating an eventual early denervation needs to be investigated. Nevertheless, data herein reported suggests that activation of A_2A_ receptors might be an important mechanism involved in the scenarios of pathology that leads to deficits in ACh release, like ALS.

Activation of A_2A_ receptors with its selective agonist CGS 21680 markedly increased the frequency of spontaneous giant events in SOD1(G93A) mice, when compared to age matched controls. Interestingly, the magnitude of this effect in the pre-symptomatic SOD1(G93A) mice (4–6 weeks old) was not different from the one observed in the WT group with 12–14 weeks old, reinforcing the “early maturation” hypothesis [7]. Modulation of Ca^2+^ dynamics by A_2A_ receptors could also be considered as an adenosine-related compensatory mechanism. In fact, it was shown that, at the mouse [19] and rat [23] neuromuscular junction, activation of A_2A_ receptors can facilitate spontaneous and evoked ACh secretion by independent mechanisms: as result of (1) an increase in cytosolic nerve terminal Ca^2+^ concentration due to release of this ion from intracellular Ca^2+^ stores or (2) by increase of extracellular Ca^2+^ entry into the terminals via L-type voltage gated Ca^2+^ channels (VGCC). Both mechanisms lead to an intracellular Ca^2+^ rise that in turn increases ACh release. Furthermore, muscle strength depends on the firing frequency and motor unit recruitment [38] and presynaptic changes in Ca^2+^ homeostasis may induce adaptations to facilitate firing frequency, specifically during high-frequency stimulation [39]. Fuchs and colleagues [40], using visually guided patch-clamp recordings in combination with single cell Ca^2+^ imaging of motor neurons throughout the complete lifespan of the SOD1(G93A) ALS mouse, reported that the pre-symptomatic motor terminals (70 days ∼7 weeks) present hyperexcitability in association with remodeling of Ca^2+^ handling.

In symptomatic mice, A_2A_ receptors modulation of both evoked and spontaneous activity was lost. Full occupancy of A_2A_ receptors by high levels of endogenous adenosine cannot account for this lack of effect, because the selective antagonist was devoid of effect on neuromuscular transmission suggesting that, A_2A_ receptors were not tonically activated by the endogenous ligand. Indeed, the experimental conditions used to evaluate changes in the quantal release of ACh (low frequency stimulation, low quantal content and muscle twitching prevented) favor reduced levels of extracellular adenosine at the endplate, since purines are released both from the nerve endings, in part together with ACh, and from the contracting muscle fibers [41], [42]. In addition, the extracellular levels of adenosine may be considerably decreased in ALS, as it occurs in other disorders of the motor endplate [42].

The loss of excitatory effect while directly activating the A_2A_ receptors with the agonist in symptomatic SOD1(G93A) mice may result from a decrease in the number and/or a decrease in the affinity of the receptor to its ligand. A_2A_ receptors expression was shown to be decreased in the spinal cord of symptomatic SOD1(G93A) animals [11]. Alterations in the transducing system operated by A_2A_ receptors may also be altered in ALS. Thus, ALS patients have increased PKA expression (the intracellular target of A_2A_ receptor activation) in the spinal cord [43], which could indicate a positive feedback response for a PKA saturation mechanism, where different proteins trigger the cAMP – PKA pathway, limiting A_2A_ receptor effects. Also immunoglobulins from ALS patients sera increased spontaneous release [44] by rendering L-type VGCC sensitive to stimuli [45], the signaling target of A_2A_ receptor activation. This could lead to abnormal interactions, resulting in impaired regulatory A_2A_ receptor recruitment of L-type VGCCs. For example, in Myasthenia gravis, a deficient A_2A_ modulation impairs recruitment of L-type VGCC rendering animals susceptible to tetanic depression [42].

Interestingly, we could observe some similarities between the symptomatic SOD1(G93A) mice (herein presented) and aged rats (70–80 weeks old; [18]) in what respects to the effect of the A_2A_ receptor selective agonist, CGS21680. In both cases there is an absence of effect of A_2A_ receptors. It remains to be clarified what are the consequences of the absence of A_2A_ receptors actions for fine-tuning of motor control and whether this relates to the age-associated or ALS-related decline in neuromuscular control. So, the reported loss of A_2A_ receptor-mediated excitatory effects in symptomatic SOD1(G93A) neuromuscular junctions could be an adaptive shift to slow motor neuron degeneration. Further studies designed to manipulate A_2A_ receptors in vivo before or after symptoms appearance may help to clarify whether A_2A_ receptors influence progression of the neuromuscular transmission deficits observed in ALS patients or if these A_2A_ receptor changes are a consequence of the disease progression. The results herein reported also pave the way for further studies designed to assess whether A_2A_ receptor changes occur in ALS patients and if so, whether they are restricted to those with SOD1 gene mutations or are present in all ALS forms.

Immuno-inflammatory processes are features present in ALS patients and in the SOD1(G93A) mouse model [46]. A_2A_ receptors have a well described immunossupressive action on immune cells [47] and their activation has proven beneficial in neuromuscular inflammatory diseases such as experimental auto-immune myasthenia gravis [48]. Schwann cells participate in adenosinergic modulation at the level of the neuromuscular junction [49] and can also participate in the modulation of immune actions [50]. A_2A_ receptors are also present on motor neurons, microglia and astrocytes helping to fine-tune motor neuron responses and participate in neuroinflammatory processes [8], [47]. A_2A_ receptors are overexpressed in lymphocytes from ALS patients, resulting in increased levels of intracellular cAMP [51], which highlights a possible role for these receptors in immunosuppressive responses in ALS. Whether the now documented A_2A_ receptor functional changes in the SOD1(G93A) also parallel with an immunological based response and relate with the previously reported A_2A_ receptor-mediated delayed onset and reduced progression of motor neuron dysfunction in this ALS model [11], awaits further investigation.

In conclusion, the work herein reported clearly documents that at the neuromuscular junction of SOD1(G93A) mice there is an exacerbation of A_2A_ receptor-mediated excitatory effects at the pre-symptomatic phase, whereas in the symptomatic phase A_2A_ receptor activation is absent. The results thus suggest that A_2A_ receptors function changes with ALS progression.
